# Emergency Teleradiology-Past, Present, and, Is There a Future?

**DOI:** 10.3389/fradi.2022.866643

**Published:** 2022-06-24

**Authors:** Anjali Agrawal

**Affiliations:** Teleradiology Solutions, Delhi, India

**Keywords:** emergency radiology, teleradiology, emergency teleradiology, international teleradiology, artificial intelligence in radiology

## Abstract

Emergency radiology has evolved into a distinct radiology subspecialty requiring a specialized skillset to make a timely and accurate diagnosis of acutely and critically ill or traumatized patients. The need for emergency and odd hour radiology coverage fuelled the growth of internal and external teleradiology and the “nighthawk” services to meet the increasing demands from all stakeholders and support the changing trends in emergency medicine and trauma surgery inclined toward increased reliance on imaging. However, the basic issues of increased imaging workload, radiologist demand-supply mismatch, complex imaging protocols are only partially addressed by teleradiology with the promise of workload balancing by operations to scale. Incorporation of artificially intelligent tools helps scale manifold by the promise of streamlining the workflow, improved detection and quantification as well as prediction. The future of emergency teleradiologists and teleradiology groups is entwined with their ability to incorporate such tools at scale and adapt to newer workflows and different roles. This agility to adopt and adapt would determine their future.

## Challenges in Emergency Radiology

Emergency radiology is an essential part of a radiology practice and has evolved into a recognized subspecialty worldwide (1). The major challenges in the field are the ever increasing imaging volumes and study complexity and increasing expectations of the referring physicians and patients, without an appropriate increase in radiologist numbers (2–4). This has led to an abysmal demand-supply mismatch, further accentuating the service gaps during after-hours or in less-desirable geographical areas, with radiologists choosing to work during their wake hours from locations of their choice (5). These deficiencies are more pronounced in domains requiring subspecialty experience^1^.

## What Is Teleradiology?

Teleradiology is a technology enabled way of radiology, wherein images acquired in one location can be transmitted over any distance for viewing at a location remote from the point of acquisition.

### Evolution

While it was used widely by the military to render diagnoses of images of the sick personnel deployed in different terrains and countries by the expert radiologists located at the base hospitals, the propelling factors in civilian settings were those of convenience and optimization of available resources. The initial reports mention the successful deployment of a link between Massachusetts General Hospital and Boston's Logan Airport, where an X-ray machine had been installed to evaluate recently landed travelers with respiratory problems and helping the physician provide his expert advice without navigating horrendous traffic for every consultation (6). The initial teleradiology systems in the eighties used camera systems or video grabbing or laser digitization of analog films for a digitized image transfer. Around the same time in the mid-eighties, PACS systems started gaining traction in radiology departments demonstrating the advantage of digital workflows (7). Progress in teleradiology remained relatively slow until the 1990s, when it became a feasible way of supporting radiology practice. Over the next few years, both the computers and internet became affordable paving way for a more widespread implementation of teleradiology (8). In the first decade of the new millennium, rapid growth took place, largely driven by the need for after-hours coverage by US hospitals, and it is in the United States that the adoption of teleradiology has been near-complete (9).

### Adoption of Teleradiology

In the wake of the US, Australia has had fairly rapid adoption of teleradiology utilization, with 67% of Australian radiologists using some form of it by 2005 (10). Europe has had a relatively slower adoption of teleradiology compared to the USA, possibly related to linguistic issues and a more regulated environment for commerce. Asia, South America and Africa have been lagging behind in the adoption and utilization of teleradiology. In India, teleradiology was reportedly first attempted at a private Imaging Center in Mumbai in 1996 (11). Proof of concept studies demonstrating the clinical feasibility of transcontinental teleradiology were conducted in Bangalore at the turn of the millennium (12, 13). While international reporting of scans from the US, Singapore, Africa, Middle East and Europe has been in practice as early as 2000 from teleradiology centers in India and other countries with high levels of accuracy and rapid report turnaround (14–16), domestic utilization of teleradiology was limited to internal coverage by radiology groups and hospitals till a few years ago. In the wake of increased awareness, rising demand for imaging support and wide penetration of broadband technologies, teleradiology has become integral to many radiology practices, with an even stronger push toward an equal share during the COVID-19 pandemic, with its offerings of radiologist availability, accessibility and efficiency, while maintaining social distancing (17, 18). Teleradiology has leveraged the advent of internet, universal adoption of digital imaging and the DICOM standard, department and enterprise level PACS, and rapid growth of computing technologies to provide a workflow balance transcending geographical boundaries. The modular separation of the different steps starting from image generation, transmission, viewing and interpretation have enabled international teleradiology with workflows connecting referring centers and radiologists in disparate regions united by radiologist qualifications and legal frameworks (19–21).

### Teleradiology and the Gartner Hype Cycle

Teleradiology has been through its growth cycle of tumultuous growth mired in controversy. It has proven its value over the last two decades, starting off as a curiosity, to a value add service in the realm of luxury, a technology set to uproot the established practice patterns and shake the professional sensibilities and the art of practice of imaging and diagnosis, to a necessity for a sustainable radiology practice in the present time (22).

## Emergency Teleradiology

### Benefits of Emergency Teleradiology

Of the many applications of teleradiology, emergency radiology consultations are the most important. The essential premise of emergency teleradiology is the need for a human being to provide accurate and timely interpretations for emergency cases where no such qualified person is locally available. This has worked well and expanded rapidly, involving radiologists from the same group stationed in different hospitals or involving external teleradiology groups within a country or internationally. The external teleradiology providers have become a significant part of emergency teleradiology enterprise, as they can offer round the clock coverage without a downtime, in parallel to the emergency room workload and imaging demands. Such an uninterrupted service is a mammoth and unaffordable task for small or medium-sized radiology groups trying to meet the on-site department requirements, administrative responsibilities of the bigger enterprise and teaching commitments. Teleradiology groups are able to garner radiologist hours from multiple full-time or part-time radiologists, irrespective of their geographical location, and provide radiology services to scale. They are able to absorb the surges from the emergency rooms and critically ill hospitalized and get the best radiologist for the imaging modality or organ system at hand. Such a spare capacity is impossible for standalone radiology groups, given the persistent radiologist shortage, increasing attrition trends and the high burnout of radiologists. Studies have established the contribution of teleradiology toward prevention of unnecessary patient transportation, unnecessary treatment, improved quality of treatment, and improved utilization of a radiologist by sending images to where he or she is located (23).

### Challenges of Emergency Teleradiology

#### Meeting Greater Expectations

While teleradiology is to a great extent able to balance the demand with supply, using a much larger work pool of experts, the fundamental problems of increasing imaging volumes, complex imaging protocols, increasing expectations of referring colleagues and patients, radiologist shortages and burnout, being faced in emergency radiology remain unaddressed. There is also an increased desire for providing a personal touch to each referral while aiming for more standardized workflows and reporting formats which aim to preserve a basic desired standard uniformly across teleradiology groups. While safeguarding quality, teleradiology groups are also under immense pressure to stick to strict turn-around time (TAT) service level agreements, more so as the studies are those of critically ill, acute stroke or major trauma, patient outcomes of which are determined by the promptness of appropriate management (24).

#### Quality Assurance of a Teleradiologist

The teleradiologist must have the necessary qualifications, accreditation, licensures and malpractice insurance coverage akin to an on-site radiologist. While the consultations rendered are legally under the purview of the jurisdiction of the site of origin of patient imaging, the radiologist should be compliant with the requirements of both the transmitting and receiving locations. The teleradiologist must also engage in continuing medical education programs and internal or external peer review or selective dual reading to maintain high quality (3, 16, 25).

#### Privacy and Security of Data

Confidentiality of patient information is maintained by staying compliant with various prevailing regulations at the source of patient data and the interpretation site such as the Health Insurance Portability and Accountability Act (HIPAA) in the US and the European Commission's Directive on Data Protection in Europe, and maintaining a secure network in the entire process of data exchange. This makes IT an essential component of a teleradiology enterprise (3).

#### Robust and Ongoing Communication

Teleradiology groups must maintain robust communication channels amongst their various support teams, radiologists and with the client hospitals or radiology groups. This in a virtual environment is challenging and requires back up methods in times of malfunctioning software, internet or occasionally power outages. Such processes must be clearly outlined and shared with the entire team such that unexpected breakdowns can be handled better without adversely affecting patient care. All critical results must be communicated in keeping with the regional guidelines and the requirements of the individual referring providers.

#### Billing for Teleradiology Services

There are different billing models based on local practice norms and regulations. Direct billing to the insurance provider may be done for internal teleradiology services. In case of external services, the referring facility bills for and collects the professional component of the bill and pays the teleradiology group a prenegotiated fee, while abiding by the antimarkup rule. Knowledge of the usual location of the teleradiologist is also required (3, 26)^2^.

## Artificial Intelligence in Emergency Teleradiology

### Need in the Emergency Setting

The need to meet greater expectations for quality, turnaround time and radiologist shortages in a virtual practice has paved the way for deployment of AI tools in different steps of the teleradiology workflow to make the processes more efficient and accurate with less human effort (27). Integration of these tools into teleradiology workflows is easier than conventional radiology practices as all available data is digital in teleradiology enterprises, extending beyond the DICOM images to data involving various practice quality metrics including patient data, hourly workloads, TATs, communication details, error rates for individual radiologists and for the group (24, 28).

### Applications in Emergency Teleradiology

The applications include automated case retrieval of relevant prior examinations eliminating the need for manual intervention involving verbal or text communication, triage of cases detected by AI tools to have critical findings warranting immediate attention and action such as intracranial hemorrhage, pneumothorax, pulmonary thromboembolism, and many more (29, 30). The automated tools can provide quantification of abnormalities and free up the radiologist for more complex cases or communication with the referring doctors or patients. Positive patient outcomes with the use of AI in detection of large vessel occlusion makes for a strong case for the utility of these tools (31). Usage of voice recognition software integrated with RIS-PACS has been a game-changer in terms of reporting speed and accuracy. The ability of tools to summarize the findings and generate impression incorporating the standard guidelines and recommendations is useful to ensure up-to-date and clear communication at all levels. The value added was noticeable during the COVID-19 pandemic (32). It has also been useful in providing structured reports catering to different populations at different levels of complexity or linguistic backgrounds. Any critical finding needs appropriate communication to the caregiver, and such automated alerts can be built into the reporting systems to avoid any slips. In large teleradiology practices with radiologists of different subspecialties and qualification degrees, automated case assignment to the best available radiologist for the given study can alleviate the load of manual intervention which can often be highly inefficient in complex matrix type teleradiology models. AI tools can also provide feedback to individual radiologists regarding their performance and point toward deficiencies, an immensely useful aid for the quality conscious enterprises^3^.

### Validation and Integration Challenges

**Figure 1:**
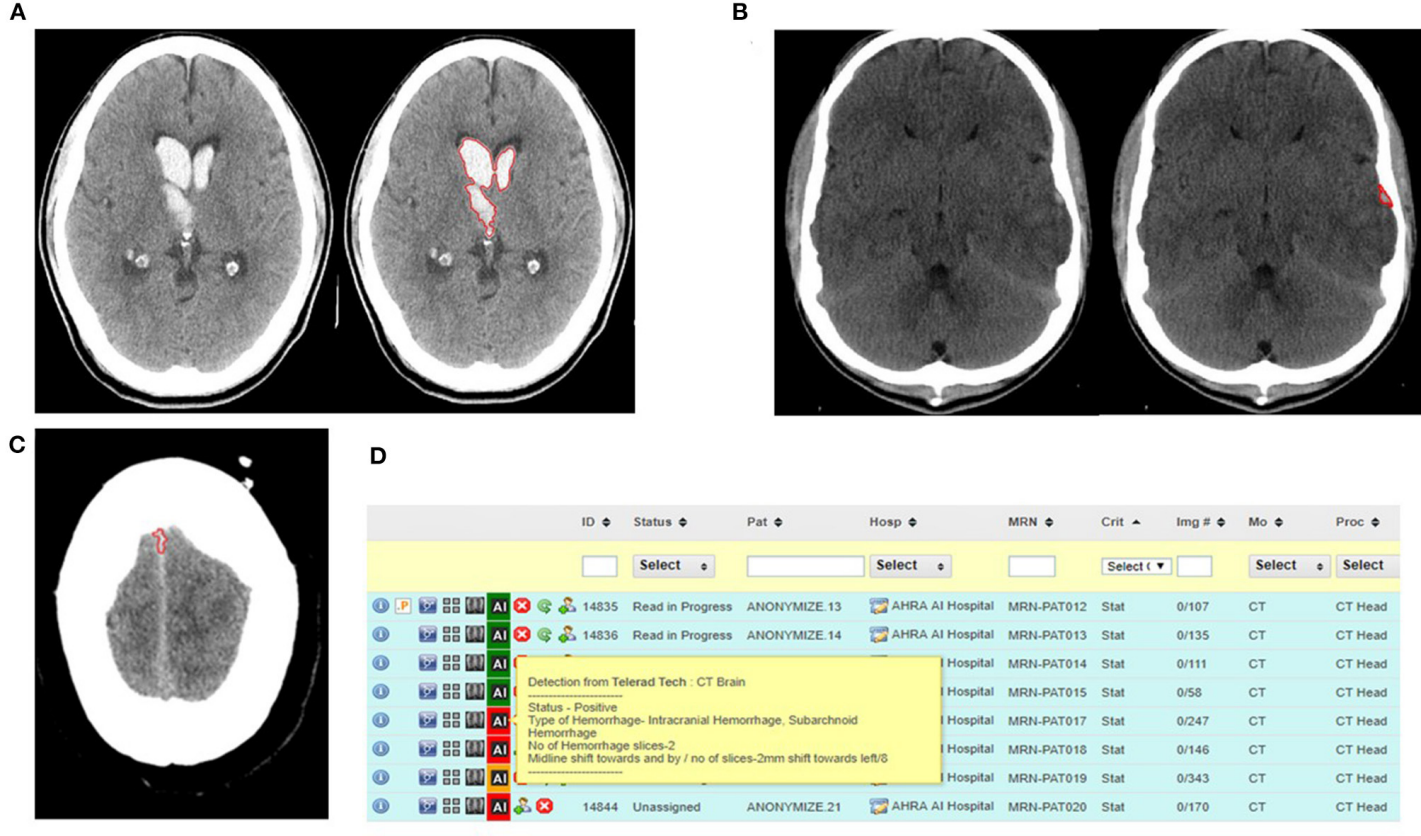
**(A)** Automated detection and segmentation of hemorrhage in the right thalamus and bilateral ventricles. **(B)** Automated detection and segmentation of extra-axial hemorrhage in the left temporal convexity. **(C)** False positive for intracranial hemorrhage with false detection of a normal hyperdense falx as hemorrhage. **(D)** Illustration of the AI tool integrated into the worklist for triage of cases with the cases positive for hemorrhage coded as red to draw radiologist's attention (RADSpa).

The evolution of such tools to the point where they are seamlessly integrated into the practice of radiology may take longer than it seemed during a phase of initial enthusiasm and partial success. An apt example is the AI tool developed in-house in my group for detection and localization of acute intracranial hemorrhage on CT head examinations (Figures 1A,B). While the tool performed fairly well in a controlled environment with a sensitivity of 97% and specificity of 85.3%, the false positive rate of 15% was too high for direct clinical use (Figure 1C). Integration into the routine workflow helped detect an occasional case of subtle intracranial hemorrhage which was missed by the radiologist, but, for the most, it was only useful in triage on an extremely busy day (Figure 1D). More often than not, the false positive alerts were irksome and distracting. The tool has undergone multiple iterations since then with improved results. This example highlights what is normal for most AI tools that underperform in live clinical environments, and therefore stringent external validations and built-in feedback loops for continued improvement are critical.

### Newer Challenges for Emergency Radiologists

With the caveats above, it is also equally clear that the inevitable growth of artificial intelligence systems, some of which are already semi-autonomous with minimal supervision such as bone age estimation tools, will put the role of radiologists in a state of flux (33). Radiologists across the board will need to embrace this tsunami of changes and disruptions. Synthesis of the various findings into a reasonable diagnosis or differential diagnosis and providing clear advice regarding further course of action would be all important. Detection of abnormalities on images would no longer be sufficient, and may come under the purview of a battery of AI tools (34). As is expected, radiologists offering a one stop shop diagnostic and therapeutic service, invested in developing special skills, providing image guided therapies, would be always desirable team members (35).

### Newer Challenges for Emergency Teleradiologists

While this new generation of emergency radiologists available on-site would still be required for emergent interventions and as active members of the emergency management teams, emergency teleradiologists face a different set of challenges, as they would have to learn to adapt and evolve in a virtual digital environment. Just as the essential premise of a human radiologist being available on-site for accurate and timely opinion was eroded by teleradiologists, now remote human teleradiologists may see competition from artificially intelligent solutions, proven to be capable of specific tasks. While replacement is unlikely, displacement into newer roles seems likely. For example, a teleradiologist may shift from a primary reader of cases to an overseer of the less experienced using approved diagnostic software, occasionally stepping in to resolve a complex case. In such a scenario, one could say that while emergency teleradiology will continue to thrive, it will be in a new avatar, very different from the one today, and would continue to offer solutions despite a scarcity of trained radiologists. Going forward, agility and adaptability toward becoming part of comprehensive digital workflows will be a critical skill for aspiring teleradiology providers. Amongst many unknowns, one foreseeable quality that will distinguish the successful teleradiologist of tomorrow will be an ability to be the overseer of artificially intelligent systems running behind the scenes, to synthesize medical information with that from the images, while also being the human face or voice that engages with other medical professionals (36). At a larger scale, teleradiology groups will transition from being managers of human radiologists to full-fledged technological solution providers actively involved in testing and validation of AI tools, and some also actively engaged in developing their own and integrating such algorithms into the larger operations and workflow management. Such larger groups equipped with AI solutions would be able to expand their reach and impact to unimaginable proportions, with favorable economics. A newer set of ethical and legal issues and guidelines would have to be in place to bring order to the newer hybrid AI assisted teleradiology practices.

Would individual practitioners and small operations come under threat? I think so. It is time to realize that at the cutting edge of technology, change is the only constant.

## Data Availability Statement

The original contributions presented in the study are included in the article/supplementary material, further inquiries can be directed to the corresponding author/s.

## Ethics Statement

Ethical review and approval was not required for this study in accordance with the local legislation and institutional requirements. Written informed consent was not required for this study in accordance with the local legislation and institutional requirements.

## Author Contributions

The author confirms being the sole contributor of this work and has approved it for publication.

## Conflict of Interest

AA is employed by Teleradiology Solutions, Delhi, India.

## Publisher's Note

All claims expressed in this article are solely those of the authors and do not necessarily represent those of their affiliated organizations, or those of the publisher, the editors and the reviewers. Any product that may be evaluated in this article, or claim that may be made by its manufacturer, is not guaranteed or endorsed by the publisher.

## Footnotes

^1^Available online at: https://www.rsna.org/news/2020/february/international-radiology-societies-and-shortage.

^2^Available online at: R3103CP.pdf (cms.gov).

^3^Available online at: https://www.vrad.com/service/technology-innovation/.
